# Mammalian carnivore occupancy is inversely related to presence of domestic dogs in the high Andes of Ecuador

**DOI:** 10.1371/journal.pone.0192346

**Published:** 2018-02-28

**Authors:** Galo Zapata-Ríos, Lyn C. Branch

**Affiliations:** 1 Department of Wildlife Ecology and Conservation, University of Florida, Gainesville FL, United States of America; 2 Wildlife Conservation Society–Ecuador Program, París, Quito Ecuador; Sichuan University, CHINA

## Abstract

Although the Andes have long been occupied by people, habitat loss, fragmentation through deforestation, and other human activities such as introduction of invasive species have increased drastically during the past century. The Ecuadorian Andes are considered a biodiversity hotspot. However, the fauna and threats to the region are poorly studied, and understanding of factors that shape the distribution of species in habitats disturbed by human activities is needed to identify and mitigate region-wide threats to wildlife. We evaluated factors associated with patterns of occurrence of Andean carnivores in landscapes of the northern Ecuadorian Andes, particularly habitat loss, fragmentation, and occupancy of domestic dogs, and determined whether thresholds occurred for these factors beyond which carnivore occurrence declined markedly. Five study areas (each 20 x 20 km) were surveyed with a total effort of 2,800 camera trap nights. Occupancies of four of the eight carnivores known from the region were best predicted by occupancy of domestic dogs rather than measures of habitat loss and fragmentation [Andean fox (*Pseudalopex culpaeus*), puma (*Puma concolor*), striped hog-nosed skunk (*Conepatus semistriatus*), and Andean bear (*Tremarctos ornatus*)]. The two largest carnivores, puma and Andean bear, demonstrated significant threshold responses to the presence of domestic dogs at two sites. Four smaller carnivores were recorded too infrequently to model occupancy, and at least two of these species appear to be in decline. The magnitude of domestic dog impacts on native species in tropical areas like the Ecuadorian Andes currently are not recognized. Results of our study indicate that small and large carnivores are in urgent need of conservation and clearly point to dogs as a significant threat to a broad range of native species.

## Introduction

Mammalian carnivores are thought to be particularly vulnerable to local extinction in disturbed landscapes because of their large home ranges, low population sizes, and direct persecution by humans [1,2]. Habitat loss and fragmentation often lead to decreased population size, increased isolation, and edge effects that increase extinction risk [3–5]. Landscape change also often is accompanied by other human disturbances such as hunting and introduction of domestic animals and other invasive species that affect carnivores [6,7]. For example, retaliation killing of carnivores that prey on livestock is common in human-dominated landscapes across the globe [8,9]. Substantial evidence also indicates that domestic dogs (*Canis lupus familiaris*) are a significant threat to wildlife [10,11]. Domestic dogs are the most abundant carnivore on Earth and have the potential to have large impacts on native carnivores because they can function as predators and competitors, as well as spread diseases [10,11,12–16]. Despite the long history of research on carnivores, particularly in temperate regions, the ecology of the vast majority of carnivore species and their responses to human-caused disturbances are poorly known, resulting in a significant gap for design of strategies to mitigate threats to these species.

In the heart of the Tropical Andes region, the Ecuadorian Andes is one of the most biologically diverse regions on Earth, and contains one of the least studied mammalian faunas in South America, including at least eight species of carnivores (e.g., [17,18]). In contrast to many lowland tropical regions where large-scale landscape change is relatively recent, the Ecuadorian Andes have long been occupied by people and, therefore, are cultural landscapes. However, habitat loss through deforestation and human development has been severe in the Ecuadorian Andes throughout the past century, and land conversion for agriculture and human population growth has further increased in recent decades [19]. Now 80% of the native habitat below 3,000 m has been converted to other uses [20,21]. Intentional killing of carnivores, primarily pumas (*Puma concolor)* and Andean bears (*Tremarctos ornatus*), because of conflict and cultural intolerance also has increased recently in some parts of the Ecuadorian Andes [22]. In addition, domestic dogs are ubiquitous throughout the region, including free-ranging dogs that are unconfined but owned or associated with human settlements, and feral dogs that are completely wild and independent of human sources of food and avoid contact with people [12,16,23]. As a result of high anthropogenic impacts and biological diversity, the Ecuadorian Andes are designated as a hotspot of endangerment for the highly endemic biota in the region [24]. However, information on Andean mammal ecology is largely anecdotal from reports and descriptions by naturalists and local hunters, and some species are only known from few museum records [24]. Thus, the status of most mammal species and their response to anthropogenic changes are unknown [16].

Our objective was to identify key factors that influence occurrence of carnivores in the fragmented landscapes of the northern Ecuadorian Andes as a basis for understanding region-wide threats to these species. At least eight species of native carnivores occur in the Ecuadorian highlands, including species at all levels of extinction risk which suggests that these species may exhibit a range of responses to human disturbance [25,26]. Differences in habitat specialization among carnivores have been proposed as an important determinant of extinction risk because species that are habitat specialists are more prone to extinction from changes in their habitats than are habitat generalists [9,27]. We hypothesized that habitat loss and fragmentation would be most important in explaining occupancy of habitat specialists, and that other factors not directly related to landscape amount and configuration, such as presence of domestic dogs, might be particularly important for habitat generalists. In addition, we examined whether thresholds values, beyond which carnivore occurrence declines markedly, occur in key explanatory factors. Thresholds are important for management because slight changes in conditions near critical thresholds could produce large changes in abundance and distribution of wildlife species [28].

## Methods

### Ethics statement

This work was carried out with support and permission of the Ministry of Environment of Ecuador (20-2009-IC-FAU-DPAP/MA). No animals were captured or sacrificed during this study, and all data were collected with camera traps. The research protocol was approved by the Non-Regulatory Animal Research System of the Institute of Food and Agricultural Sciences, University of Florida (IFAS/ARC#015-08WEC).

### Study areas and species

**Fig 1:**
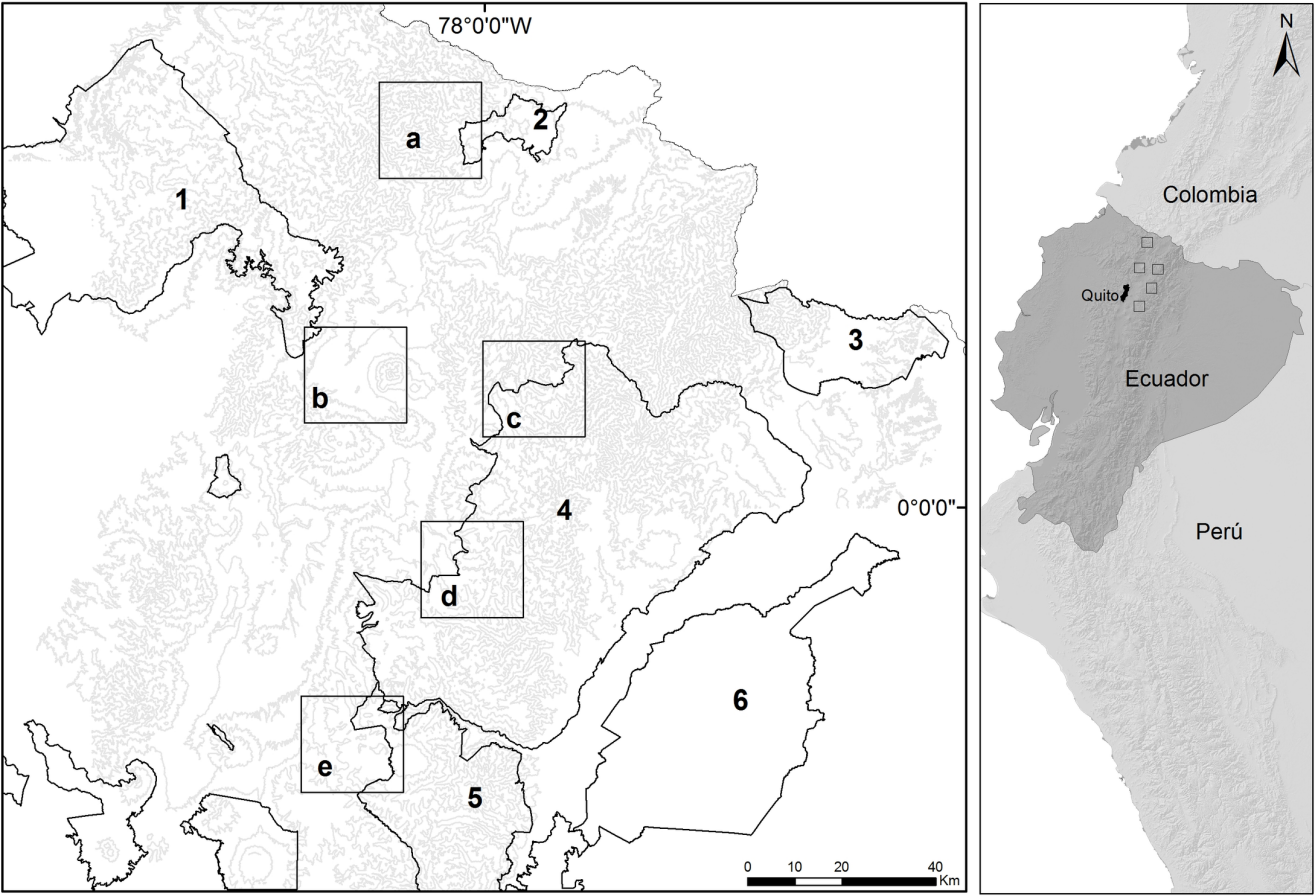
**Map of the northern Ecuadorian Andes showing the location of the study areas** (a, El Morán; b, Fuya-Fuya; c, Filo Curiquingue; d, San Marcos; and e, Guaytaloma), and protected areas (1, Cotacachi-Cayapas Ecological Reserve; 2, El Ángel Ecological Reserve; 3, Cofán Bermejo Ecological Reserve; 4, Cayambe-Coca National Park; 5, Antisana Ecological Reserve; and 6, Sumaco-Napo-Galeras National Park).

The study region, located in the northern Andes of Ecuador (Fig 1), is a mosaic of protected areas (e.g., Cayambe-Coca National Park, El Ángel Ecological Reserve, Antisana Ecological Reserve), agricultural areas, and indigenous territories (Andean Kichwas) that encompass snow-capped volcanoes, páramos (high altitude grasslands and shrublands), and Andean forests dominated by tree line species (e.g., *Polylepis* spp. and *Gynoxis* spp.) [29]. Land-cover classes such as agriculture, exotic tree plantations (*Pinus radiata* and *Eucalyptus globulus*), native forest patches, native shrubland and grassland patches are interspersed with human settlements, roads, wetlands, and streams [30]. Climate is highly aseasonal, with a mean of 2000 mm annual precipitation, and an average monthly temperature of 14 °C [31].

Five study areas ranging from 2800–3800 m above sea level (each 20 x 20 km) were sampled in this study (Fig 1). El Morán (00°47’58”N, 78°00’06”W) and Fuya-Fuya (00°08’06”N, 78°18’31”W) are located along the western Andean mountain range, and Filo Curiquingue (00°11’32”N, 77°59’55”W), San Marcos (00°06’19”N, 77°57’43”W) and Guaytaloma (00°19’31”S, 78°08’53”W) are located along the eastern mountain range. The percentage of native vegetation remaining in the five study areas ranges from 56% in Fuya-Fuya to 73% in Filo Curiquingue [32] and comprises Andean forests, páramo grasslands, and páramo shrublands. Kichwa communities are located along the borders of protected areas, and base their livelihood on agriculture and livestock [21,33], which results in a fragmented landscape with native vegetation (forest, shrubs, and grasslands) embedded in a matrix of livestock grazing areas (mainly cows and sheep) and agricultural land (mainly corn and potatoes).

Eight species of native carnivores occur in the highlands of the northern Ecuador: Pampas cat (*Leopardus colocolo*), puma (*Puma concolor*), Andean fox (*Pseudalopex culpaeus*), Andean bear (*Tremarctos ornatus*), Colombian weasel (*Mustela felipei*), long-tailed weasel (*Mustela frenata*), striped hog-nosed skunk (*Conepatus semistriatus*), and mountain coati (*Nasuella olivacea*). Two of these species (long-tailed weasel, striped hog-nosed skunk) are small (<10 kg) habitat generalists. Three species are small habitat specialists (pampas cat–high altitude grasslands, mountain coati–Andean forest, Colombian weasel—riparian habitats in Andean forest). The Colombian weasel and the mountain coati are endemic to the Tropical Andes (~2000–5000 m above sea level). Two other species (puma, Andean fox) are large (>10 kg) habitat generalists, and the Andean bear is a large habitat specialist primarily found in high altitude grasslands and cloud forest [22,34–37]. Ranges of these eight carnivores coincide with areas of high human population density in Ecuador [25]. According to the IUCN Red List of Threatened Species [38], the Andean bear and the Colombian weasel are Vulnerable, and the mountain coati is Data Deficient. At the national level, the Ecuadorian Red List of Endangered Mammals [26] categorizes the Andean bear as Endangered; the pampas cat, puma, Andean fox, and mountain coati as Vulnerable; and the Colombian weasel as Data Deficient.

### Carnivore surveys

Between April 2009 and July 2010, we conducted presence-absence surveys of native Andean carnivores and domestic dogs using camera traps (Bushnell Trail Sentry STD, Bushnell Corp., Overland Park, KS, USA). We subdivided each study area (400 km^2^) into 16 cells of 25 km^2^; and in each cell, we randomly placed seven camera traps at least 1000 m apart (for a total of 112 camera traps in each study area). We chose random sites rather than targeting specific features that might be used by carnivores (e.g., trails) in order to avoid potential biases associated with differential use of these features by different species. When a randomly selected site was inappropriate (e.g., located on a rocky cliff), we selected the closest appropriate site to place the camera. We used commercially available carnivore urine as an attractant (http://www.predatorpee.com), and we randomly chose red fox or bobcat urine for each camera trap. To create capture histories for each species, we employed the camera traps for five consecutive days (560 trap-nights per study area) and recorded detection/non-detection for each day for each camera.

### Environmental variables in occupancy models

To identify environmental factors related to the presence of native carnivores and domestic dogs, we developed a series of occupancy models with variables that represented habitat loss and fragmentation and other human disturbance, including occupancy of dogs for models of native carnivores (Table 1). Metrics for loss and fragmentation of native habitat were assessed at three spatial scales: 1) site—radial distance of 20 m from the camera trap; 2) home range scale—radial distance of 1000 m from the camera trap for small species (<10 kg), and 5000 m for large species (>10 kg); and 3) landscape scale—400-km^2^ study area. We included distance of camera traps from the nearest house and road as surrogates for other human disturbance (e.g., noise and traffic) that might negatively impact carnivores. The presence of dogs also often is inversely related to distance from human habitation [39]. However, in our study region, relatively pristine areas (e.g., national parks) contain populations of feral dogs that are not associated with humans [16]. Thus we did not expect to encounter a simple inverse relationship between presence of dogs and distance from houses and roads, and we examined this relationship in occupancy models for dogs. Where subsistence hunting occurs, hunting pressure also often is greatest near human settlements [40,41] and, thus, correlated with other human disturbance. However, local people throughout the northern Ecuadorian Andes do not hunt game for subsistence but rather rely on domestic animals for protein. Hunting of carnivores (primarily Andean bears and pumas) in retaliation for livestock depredation has been reported in the Ecuadorian Andes [22] but we found no evidence of hunting in our study areas (e.g., no signs of hunters or photographs of hunters in camera traps) and, thus, did not consider this factor in our assessment.

**Table 1:** Variables for habitat loss and fragmentation and other aspects of human disturbance measured at five sites in the Ecuadorian Andes where occupancy of carnivores was modeled, explanatory set of variables for the models, range of values for variables, scale at which the variables were measured, and source of data. Asterisks show variables retained for occupancy analyses.

Variable	Acronym	Set^1^	Range	Scale^2^	Source of raw data
Distance to nearest patch of native vegetation > 1 km^2^	DNPNV	Frag	0.45–16.78 km	L	Maps [32]
Proportion of native habitat (plot radius of 1,000 and 5,000 m)	PRONH*	Hab	0.17–0.93	H	FRAGSTATS 3.0 [42]
Number of patches (plot radius of 1,000 and 5,000 m)	NUMPAT*	Frag	3–11	H	FRAGSTATS 3.0 [42]
Total edge (plot radius of 1,000 and 5,000 m)	TOTEDG	Frag	0.65–3.74 km	H	FRAGSTATS 3.0 [42]
Land cover (native and non-native vegetation)	LANCOU*	Hab	Six categories	S	Ground assessment
Patch size (if camera trap located in native vegetation)	PATSIZ	Frag	365–27 000 000 m^2^	L	FRAGSTATS 3.0 [42]
Distance to nearest native patch (if camera in non-native vegetation)	DISNPAT	Frag	0.074–1.69 km	S	Maps [32]
Distance to nearest road	DISROA*	Other	0.023–8.34 km	L	Maps [43]
Distance to nearest house	DISHOU*	Other	0.19–11.28 km	L	Maps [43]
Occupancy rates of domestic dogs	DOG*	Other	0.11–0.86	S	Camera traps

^1^Frag (fragmentation), Hab (habitat loss), Other (other disturbance).

^2^L (landscape, 400-km^2^ study area), H (home range, 1000-m radial distance for small species, 5000-m for large species), S (site, 20-m radial distance from camera trap).

Environmental variables were measured in the field and from existing GIS layers. We determined land-cover type (native and non-native vegetation) at each camera site and extracted other variables for habitat loss and fragmentation and distance to the nearest house or road (Table 1) from digital maps (scale 1:50,000) of vegetation and topographic features [32,42] with FRAGSTATS 3.0 software [43]. Highly correlated variables (r > 0.5) for habitat loss and fragmentation were omitted from occupancy analyses (Table 1, S1 Table). For measures of habitat loss, we used proportion of habitat that was native (PRONH) at the home range scale and six categories of land cover (LANCOU) at the site scale (Table 1). Native vegetation included grass páramo, shrub páramo and Andean forest, and non-native vegetation included pasture with introduced grasses, crops and forest plantations. For an index of fragmentation, we used number of patches of native vegetation (NUMPAT). Occupancy rates of domestic dogs (DOG), modeled from the camera trap data, were included as a measure of presence of this species [44].

### Modelling approach

We evaluated the influence of habitat loss and fragmentation, presence of dogs, and other human disturbance on carnivore occupancy (*Ψ*) and detectability (*p*) using two approaches. First, we modeled site-based occupancy for each study area with single-species, single-season occupancy models [45]. For each species, we modeled detection probability using the global model of occupancy, and then the best detection models were used with occupancy models [46,47]. Detection probability of both Andean carnivores and dogs was modeled as a function of lunar cycle (MOON) and the two types of attractants (red fox and bobcat urine, LURE). Moon phase affects carnivore behavior and activity because moon brightness alters detectability of both predators and prey [48,49]. Use of attractants in carnivore surveys typically increases detection probability of species of interest, and generally does not affect movement patterns, abundance or temporal activity patterns [50,51]. For detectability and occupancy, we included an intercept model with no covariates among the set of candidate models, and, for each species and site, we constructed occupancy models with all single predictor variables and a model with an interactive term for habitat loss and fragmentation (PRONH * NUMPAT). Values of the Akaike Information Criterion corrected for small sample size (AICc) were used to rank candidate models [52]. We assessed the fit of our global models with a goodness-of-fit test based on Pearson *X*^2^ statistic and bootstrapping [53]. Occupancy modeling was performed with the PRESENCE 3.1 software [54].

We also constructed a single season, two-species model, as a complementary analysis, to evaluate whether occurrence of native carnivores is related to presence of domestic dogs [55,56]. We estimated two parameters: ϕ, a ratio of how likely domestic dogs and native carnivores are to co-occur at a given site compared to what would be expected if they were distributed independently; and δ, a ratio of how likely domestic dogs and native carnivores are to be detected at a site occupied by both species compared to the expected detection if detection of the two species were independent [56]. When species occur independently, ϕ ≈ 1. When species co-occur less often than expected, ϕ < 1, and when species co-occur more often than expected, ϕ > 1. Likewise, when δ ≈1, detection of the two species is independent, δ < 1 observers are less likely to detect one species if the other is detected at the site, and δ >1 observers are more likely to detect one species if the other is detected [55,56]. Estimates of ϕ and δ for all species in each site were based on null models without covariates. The two-species modeling was performed also with PRESENCE 3.1 software [54].

We tested for lack of independence among sampling units due to spatial autocorrelation [57] with the weighted correlation coefficient of Moran (Moran’s *I*) in ArcView 3.2a [58,59]. Spatial autocorrelation was not evident for any species included in occupancy analyses (Moran’s *I* range = -0.18–0.34 for all species across sites; all Z-scores between -1.96 and 1.96). To evaluate the existence of potential thresholds in the responses of Andean carnivores to the variables identified as most strongly related to occupancy (i.e., distance to roads, occupancy rates of domestic dogs), we applied generalized piecewise linear regression models to the occupancy rates of carnivores with the segmented package of R [60]. This type of model identifies existing discontinuities in the data in the form of break points [61].

## Results

Domestic dogs were common in all five study areas. The Andean fox and striped hog-nosed skunk, which occur in a broad array of native and disturbed habitats, were the most commonly detected native carnivores (S2 Table) and the only species recorded frequently in all study areas. The Andean bear and puma were recorded in three and four study areas, respectively, but we only had enough data to construct models for Filo Curiquingue and San Marcos. The long-tailed weasel and mountain coati were detected only occasionally in two study areas (Filo Curiquingue and San Marcos), and the pampas cat and Colombian weasel were not detected. These four species were not included in analyses.

### Occupancy and detectability of domestic dogs

Mean estimated occupancy (*Ψ^*) for dogs was greater than 0.50 for all study areas and most strongly related to distance to houses and roads (Tables 2 and 3). However, the relationship between presence of dogs and these environmental predictors varied across sites. At two sites (Filo Curiquingue and Fuya-Fuya), occupancy was inversely related to distance to houses and distance to roads (Table 3). In contrast, the occupancy patterns of dogs at the remaining three sites increased with distance from roads and houses. Detection probability of domestic dogs was related to the lunar cycle in Filo Curiquingue and Guaytaloma (*w*+ = 0.69 and 0.87; β = 1.03 and 1.27 respectively) and remained constant at other sites (Table 2). The type of carnivore urine used as a lure had no effect on detection of domestic dogs.

**Table 2:** Top three models (occupancy, *Ψ*; detectability, *p*) for domestic dogs. DISROA, distance to nearest road; MOON, lunar cycle; LANCOU, land cover; DISHOU, distance to nearest house; NUMPAT, number of patches.

Study area	Models	*K*	AICc	ΔAICc	*w*_*i*_
Filo Curiquingue	*Ψ*(DISROA) *p*(MOON)	4	481.20	0.00	0.39
	*Ψ*(LANCOU) *p*(MOON)	4	483.14	1.94	0.27
	*Ψ*(DISHOU) *p*(MOON)	4	483.74	2.54	0.17
San Marcos	*Ψ*(DISHOU) *p*(.)	3	498.92	0.00	0.37
	*Ψ*(DISROA) *p*(.)	3	501.11	2.19	0.21
	*Ψ*(LANCOU) *p*(.)	3	501.11	2.19	0.21
Guaytaloma	*Ψ*(DISROA) *p*(MOON)	4	488.72	0.00	0.41
	*Ψ*(DISHOU) *p*(MOON)	4	490.81	2.09	0.23
	*Ψ*(NUMPAT) *p*(MOON)	4	491.11	2.39	0.23
El Morán	*Ψ*(DISHOU) *p*(.)	3	495.48	0.00	0.46
	*Ψ*(DISROA) *p*(.)	3	497.68	2.20	0.22
	*Ψ*(LANCOU) *p*(.)	3	498.32	2.84	0.14
Fuya-Fuya	*Ψ*(DISHOU) *p*(.)	3	485.62	0.00	0.38
	*Ψ*(DISROA) *p*(.)	3	487.81	2.19	0.29
	*Ψ*(NUMPAT) *p*(.)	3	488.96	3.34	0.11

**Table 3:** Estimated β coefficients for the top ranked models of occupancy and mean estimated occupancy (*Ψ^* ± 95% CI) for domestic dogs. DISROA, distance to nearest road; DISHOU, distance to nearest house.

Study area	*Ψ* top ranked	DISROA	DISHOU	*Ψ^*	95% CI
	models	β (SE)	β (SE)		
Filo Curiquingue	*Ψ*(DISROA)	-1.05 (0.22)	---	0.53	0.47–0.58
San Marcos	*Ψ*(DISHOU)	---	0.97 (0.32)	0.64	0.59–0.68
Guaytaloma	*Ψ*(DISROA)	0.46 (0.13)	---	0.71	0.66–0.75
El Morán	*Ψ*(DISHOU)	---	1.24 (0.43)	0.73	0.68–0.77
Fuya-Fuya	*Ψ*(DISHOU)	-0.69 (0.24)	---	0.69	0.64–0.73

### Occupancy and detectability of Andean carnivores

Models produced by both modeling approaches were highly congruent and indicate that occupancy rates of domestic dogs are important predictors of occupancy of the four Andean carnivores. Therefore, we present results of the single-species models in detail here (Tables 4 and 5), and results of the two-species models in S3 Table. Except for one species and site (puma in Filo Curiquingue), the model with occupancy of domestic dogs (DOG) was the top single-species model, and this model was substantially better at predicting Andean carnivore occupancy than models with variables for habitat loss and fragmentation, distance to nearest house, or distance to nearest road (Tables 4 and 5). In Filo Curiquingue, the most important variable for explaining puma occupancy was distance to nearest road (Table 4). Detection probability of two Andean carnivores (Andean fox and striped hog-nosed skunk) was related to the lunar cycle (range *w*+ = 0.64–0.83; β = 0.78–1.42), and detection probabilities were constant for the puma and Andean bear. The type of carnivore urine used as a lure had no effect on species detection. Goodness-of-fit tests did not indicate lack of fit in our global models (*X*^2^ ≥ 12.31; *p* ≥ 0.59 for all species and sites).

**Table 4:** Top three models (occupancy, *Ψ*; detectability, *p*) for Andean carnivores. DISROA, distance to nearest road; DOG, occupancy rates of domestic dogs; PRONH, proportion of native habitat; DISHOU, distance to nearest house; MOON, lunar cycle.

Species/Study area	Models	*K*	AICc	ΔAICc	*w*_*i*_
**Puma**					
Filo Curiquingue	*Ψ*(DISROA) *p*(.)	3	472.91	0.00	0.26
	*Ψ*(DOG) *p*(.)	3	474.82	1.91	0.21
	*Ψ*(PRONH) *p*(.)	3	475.87	2.96	0.16
San Marcos	*Ψ*(DOG) *p*(.)	3	467.53	0.00	0.36
	*Ψ*(DISHOU) *p*(.)	3	470.52	2.99	0.26
	*Ψ*(DISROA) *p*(.)	3	470.84	3.31	0.09
**Andean fox**					
Filo Curiquingue	*Ψ*(DOG) *p*(MOON)	4	437.26	0.00	0.41
	*Ψ*(DISHOU) *p*(MOON)	4	439.65	2.39	0.23
	*Ψ*(PRONH) *p*(MOON)	4	439.82	2.56	0.15
San Marcos	*Ψ*(DOG) *p*(MOON)	4	426.49	0.00	0.38
	*Ψ*(DISHOU) *p*(MOON)	4	428.52	2.03	0.26
	*Ψ*(DISROA) *p*(MOON)	4	428.96	2.47	0.16
Guaytaloma	*Ψ*(DOG) *p*(MOON)	4	439.03	0.00	0.37
	*Ψ*(DISROA) *p*(MOON)	4	441.36	2.33	0.23
	*Ψ*(DISHOU) *p*(MOON)	4	443.81	4.78	0.17
El Morán	*Ψ*(DOG) *p*(MOON)	4	467.03	0.00	0.39
	*Ψ*(DISHOU) *p*(MOON)	4	469.94	2.91	0.24
	*Ψ*(DISROA) *p*(MOON)	4	469.99	2.96	0.17
Fuya-Fuya	*Ψ*(DOG) *p*(MOON)	4	442.67	0.00	0.42
	*Ψ*(DISHOU) *p*(MOON)	4	444.75	2.08	0.26
	*Ψ*(DISROA) *p*(MOON)	4	445.51	2.84	0.15
**Andean bear**					
Filo Curiquingue	*Ψ*(DOG) *p*(.)	3	422.23	0.00	0.37
	*Ψ*(DISROA) *p*(.)	3	424.69	2.46	0.26
	*Ψ*(PRONH) *p*(.)	3	424.94	2.71	0.13
San Marcos	*Ψ*(DOG) *p*(.)	3	440.49	0.00	0.38
	*Ψ*(DISHOU) *p*(.)	3	442.45	1.96	0.25
	*Ψ*(DISROA) *p*(.)	3	442.52	2.03	0.17
**Striped hog-nosed skunk**					
Filo Curiquingue	*Ψ*(DOG) *p*(MOON)	4	399.85	0.00	0.39
	*Ψ*(DISROA) *p*(MOON)	4	402.15	2.30	0.24
	*Ψ*(DISHOU) *p*(MOON)	4	402.71	2.86	0.14
San Marcos	*Ψ*(DOG) *p*(MOON)	4	399.29	0.00	0.34
	*Ψ*(DISHOU) *p*(MOON)	4	401.15	1.86	0.25
	*Ψ*(PRONH) *p*(MOON)	4	402.23	2.94	0.15
Guaytaloma	*Ψ*(DOG) *p*(MOON)	4	494.09	0.00	0.42
	*Ψ*(DISHOU) *p*(MOON)	4	496.18	2.09	0.21
	*Ψ*(DISROA) *p*(MOON)	4	497.29	3.20	0.13
El Morán	*Ψ*(DOG) *p*(MOON)	4	481.57	0.00	0.37
	*Ψ*(DISHOU) *p*(MOON)	4	483.01	1.44	0.24
	*Ψ*(DISROA) *p*(MOON)	4	483.70	2.13	0.16
Fuya-Fuya	*Ψ*(DOG) *p*(MOON)	4	488.99	0.00	0.39
	*Ψ*(DISHOU) *p*(MOON)	4	491.15	2.16	0.22
	*Ψ*(DISROA) *p*(MOON)	4	492.31	3.32	0.15

**Table 5:** Estimated β coefficients for the top ranked models of occupancy and mean estimated occupancy (*Ψ^* ± 95% CI) for Andean carnivores. DISROA, distance to nearest road; DOG, occupancy rates of domestic dogs.

Species/Study area	*Ψ* top ranked	DISROA	DOG	*Ψ^*	95% CI
	models	β (SE)	β (SE)		
**Puma**					
Filo Curiquingue	*Ψ*(DISROA)	1.64 (0.37)	---	0.28	0.23–0.32
San Marcos	*Ψ*(DOG)	---	-1.59 (0.37)	0.22	0.16–0.29
**Andean fox**					
Filo Curiquingue	*Ψ*(DOG)	---	-1.18 (0.45)	0.46	0.41–0.51
San Marcos	*Ψ*(DOG)	---	-1.03 (0.32)	0.31	0.26–0.35
Guaytaloma	*Ψ*(DOG)	---	-1.44 (0.26)	0.37	0.32–0.42
El Morán	*Ψ*(DOG)	---	-1.29 (0.41)	0.34	0.29–0.38
Fuya-Fuya	*Ψ*(DOG)	---	-0.87 (0.31)	0.29	0.24–0.34
**Andean bear**					
Filo Curiquingue	*Ψ*(DOG)	---	-1.61 (0.43)	0.26	0.19–0.35
San Marcos	*Ψ*(DOG)	---	-1.36 (0.29)	0.19	0.13–0.27
**S. hog-nosed skunk**					
Filo Curiquingue	*Ψ*(DOG)	---	-0.93 (0.21)	0.51	0.46–0.55
San Marcos	*Ψ*(DOG)	---	-1.37 (0.28)	0.47	0.43–0.52
Guaytaloma	*Ψ*(DOG)	---	-1.15 (0.33)	0.42	0.37–0.46
El Morán	*Ψ*(DOG)	---	-0.68 (0.34)	0.48	0.44–0.53
Fuya-Fuya	*Ψ*(DOG)	---	-0.99 (0.27)	0.45	0.41–0.49

### Thresholds

**Fig 2:**
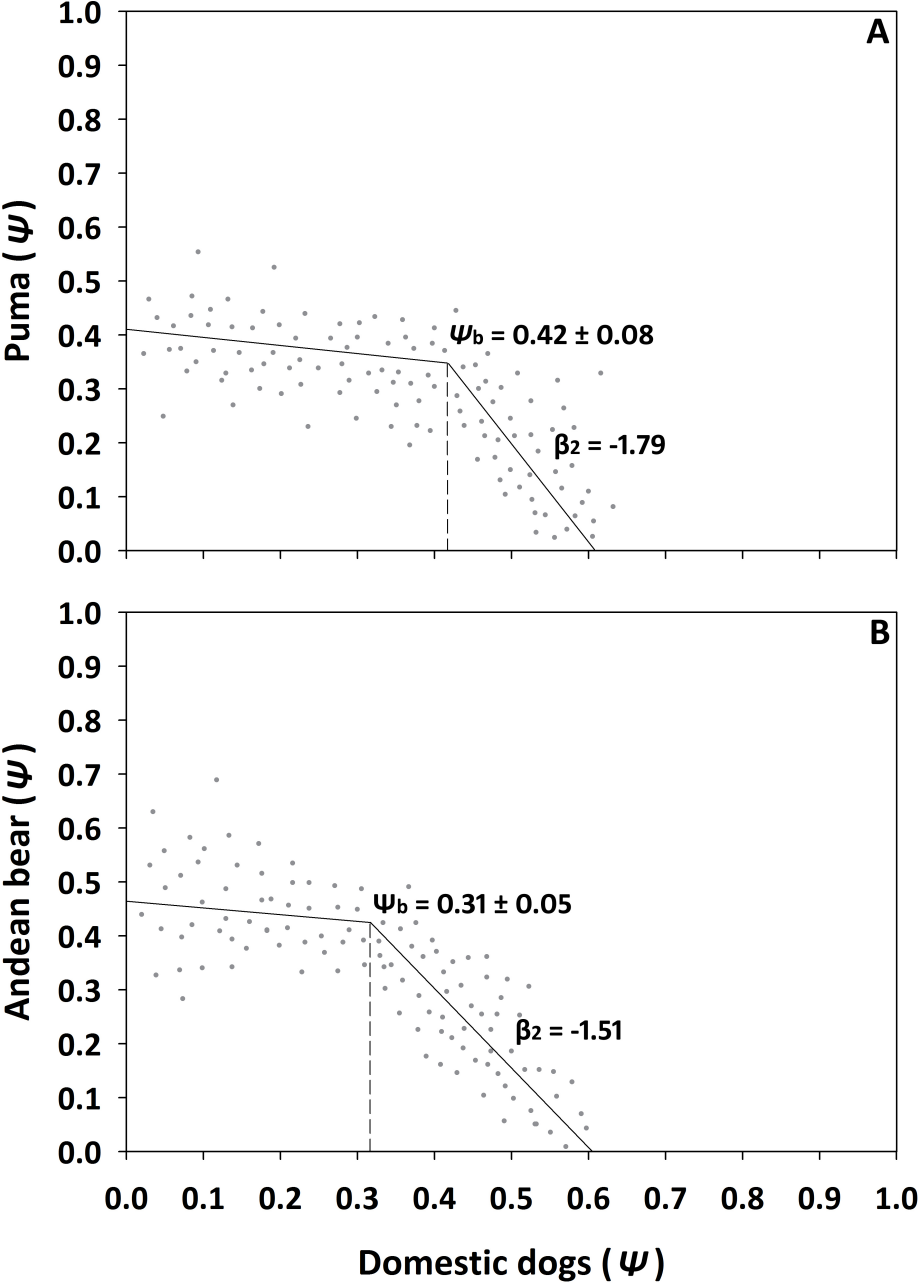
Threshold responses of A) puma and B) Andean bear to occupancy rates of domestic dogs in San Marcos and Filo Curiquingue, respectively (*Ψ*_b_, occupancy of domestic dogs at breakpoint ± CI 95%; β_2_, threshold effect).

Evidence for threshold responses by native carnivores to the most important variables was limited to responses by pumas and Andean bears at one site each (Fig 2). In San Marcos, occupancy of pumas declined sharply when occupancy of domestic dogs reached 0.42 (± 0.08 95% CI, *p* = 0.03) and, in Filo Curiquingue, occupancy for Andean bears declined when occupancy of dogs reached 0.31 (± 0.05, *p* = 0.04). No other threshold responses were detected for these species or the Andean fox and striped hog-nosed skunk (*p* > 0.05).

## Discussion

Current occupancy of four native Andean carnivores, including three habitat generalists (puma, Andean fox, and striped hog-nosed skunk) and one specialist (Andean bear), was best predicted by the presence of domestic dogs rather than habitat loss and fragmentation. These models provide significant evidence that both free-ranging and feral dogs negatively influence occupancy of Andean carnivores. Dogs affect populations of native wildlife by direct predation, through exploitative competition (asymmetric competitive abilities in obtaining limited resources), interference competition (interactions such as spatial exclusion, harassment, and intraguild predation), and by acting as vectors of disease such as canine distemper, parvovirus, and rabies [10,12,13,15,16]. Free-ranging domestic dogs usually occur in close proximity to human-dominated areas, rely heavily on human sources of food for subsistence, and do not respond numerically to potential declines of native prey [39,62,63]. Thus, free-ranging domestic dogs represent a substantial threat to Andean carnivores and other native species because they have the potential to maintain predation pressure and competition levels as native prey and native carnivores decline. Models for our most central study sites (Filo Curiquingue and Fuya-Fuya) provide support for this scenario, as occupancy of domestic dogs declined as distance from roads and houses increased, and occupancy of native carnivores was negatively related to the presence of dogs. In contrast, at remaining sites in the north and south of our study region, the negative relationship between occupancy of dogs and native carnivores was maintained but occupancy of domestic dogs increased with distances from houses and roads, likely reflecting the presence of feral dogs in these study sites. Local people report observations of feral dogs in these areas. Also, camera trapping studies in Cayambe-Coca National Park (Fig 1) in undisturbed habitats with and without feral dogs documented reduced abundance of medium and large mammals and changes in their activity patterns where feral dogs were present [16]. The results we report here suggest that the impacts of feral dogs on wildlife are widespread in the Ecuadorian highlands and, similarly, that free-ranging dogs are a significant threat.

Human-induced forest destruction and fragmentation of habitats are major drivers of biodiversity loss worldwide [3–5], and we expected these to be the key factors in explaining occupancy patterns of some Andean carnivores, particularly habitat specialists. Lack of support for these processes as the major drivers of occupancy patterns may have occurred for several reasons. Habitat loss and fragmentation may not strongly affect some Andean carnivore species because the landscape always has been patchy, comprising forest and shrub patches within a grassland matrix [29], and some pastures with introduced grasses resemble structure of native habitats. Also, habitat loss and fragmentation may have resulted in decline and loss of species in the past and only the most resistant species are widespread today and modeled in this study. Half of the native carnivore species that should occur in the study region were not detected in our study (pampas cat and Colombian weasel) or were recorded very rarely (long-tailed weasel and mountain coati). With the exception of the long-tailed weasel, all of these species are habitat specialists. In contrast, three of the four species for which we obtained sufficient data to model (puma, Andean fox, and striped hog-nosed skunk) are generalist species that traverse and forage in a wide variety of vegetation types both native and non-native [34,64–66], and the Andean bear, which specializes on high altitude grasslands, is capable of long distance movements between resource-rich patches of native habitat [67,68].

Identification of thresholds in species responses to disturbance has been recognized as potentially important for conservation and management actions [69–71]. However, as in our study, the existence of thresholds has not been consistently supported by empirical evidence [72–75]. Occupancy of pumas and bears exhibited a strong decline when the probability of occupancy by dogs reached about 0.40 and 0.30, respectively, at two sites in our study, but we did not find evidence of threshold responses at other sites or among other species of Andean carnivores although occupancy of dogs surpassed these thresholds at all sites. The reasons for inconsistencies in thresholds at our sites are unknown but could be related to a myriad of species-specific and site-specific factors, such as defense mechanisms of native species, ranging behavior of domestic dogs, and habitat that influences detection of native species [9,12,76,77].

Although most native habitat below 3,000 m of the high Andes of Ecuador has been converted to other land uses [20,21], no detailed accounts exist regarding the history of changes in the amount of habitat or configuration of the landscape or changes in the mammalian fauna [21]. Therefore, we do not know whether smaller carnivores in this region have always occurred naturally in very low population densities or whether their current status results from major declines prior to our study, and whether some species were under-sampled in this study. The long-tailed weasel is thought to be relatively common in the study region (though no population data are available) and is capable of traversing and foraging in disturbed habitats [25,78]. Weasels communicate with scent marking [79,80]. The limited number of photographs we obtained for this species could result from use of carnivore urine as an attractant. In contrast to the long-tailed weasel, the Colombian weasel is considered an extremely rare species and was only first described in 1978 [81]. The geographic range and population status of this species are unknown, but the species is reported to specialize on riparian areas within Andean forest which were not specifically targeted in our study [26,37]. Local residents indicated that the pampas cat once was widespread in our study region but numbers have decreased dramatically in the last 10 to 15 years. Camera traps are effective in detecting small cats and should have recorded this species if it were present in our study sites [48,82]. The few published studies that have examined the mountain coati also suggest that mountain coatis occur in very low population densities, present a patchy distribution, mostly limited to Andean forest in well preserved areas, and that this species rarely occurs in disturbed and fragmented areas like our study sites [83,84]. In the tropical Andes most conservation-related research on carnivores has focused on the Andean bear (e.g., [36,67]), but clearly smaller carnivores are highly vulnerable, much less known, and deserve urgent attention.

### Conservation implications and conclusions

Results of this study and our previous work in undisturbed habitat in Cayambe-Coca National Park [16] indicate that small, as well as large, carnivores are in critical need of conservation in the Ecuadorian Andes and clearly point to dogs as a significant threat to a broad range of wildlife species. Dog owners in rural areas in tropical regions like the Ecuadorian Andes commonly abandon dogs and neglect to feed and vaccinate them. Under these circumstances, domestic dogs often become free-ranging, or even feral within protected areas at the margins of development [85].

Unlike habitat loss and fragmentation, occurrence of domestic dogs is not easily mapped at large spatial scales and as a result, the extent and intensity of domestic dog impacts on native species are unknown in most regions, despite the fact that domestic dogs are ubiquitous throughout the world and far outnumber all other native canid species combined [10,12,13,16,86–88]. For example, no population size estimates are available for dogs in the Ecuadorian Andes, and the time period in which dogs became a problem for wildlife in the region is unknown. However, human and dog population sizes generally are strongly correlated [11,13]. The human population in the Ecuadorian Andes (approximately 7 million) is the densest (100 people/km^2^) in the entire Andean region of South America [89] and, as a result, the Ecuadorian Andes may have a particularly large population of poorly managed domestic dogs. However, at least 40 million people, and their dogs, live in the Tropical Andes, laying the foundation for a widespread problem [90]. Based on our findings, in the last five years the Ministry of the Environment and other organizations in Ecuador have initiated a broad-based management program to eliminate feral dogs from protected areas and control dog populations in other rural areas (including domestic dog vaccination and sterilization programs, and creation of new regulations for managing and controlling domestic animals in buffer areas surrounding protected areas). Such efforts deserve recognition and could serve as a model to address impacts of dogs on wildlife and wilderness in other regions where human activities are widespread in the landscape and systematic management of domestic dogs is not common.

## Supporting information

S1 Table Correlation coefficients among the variables measured for occupancy models of Andean carnivores and domestic dogs. (XLS)

S2 Table Number of independent detections, relative abundance index (RAI, number of independent detections/100 trap-nights) and mean latency to initial detection (LTD, trap-nights) of six species of native carnivores of the Ecuadorian Andes. (XLSX)

S3 Table Parameter estimates and standard error of the two-species occupancy model for domestic dogs and native carnivores. (XLSX)

## References

[1] Sunquist M, Sunquist F. Changing landscapes: consequences for carnivores In: Gittleman JL, Funk SM, Macdonald DW, Wayne RK, editors. Carnivore Conservation. Cambridge: Cambridge University Press; 2001 pp. 399–418.

[2] Crooks KR. Relative sensitivities of mammalian carnivores to habitat fragmentation. Conserv Biol. 2002; 16(2): 488–502.

[3] Saunders DA, Hobbs RJ, Margules CR. Biological consequences of ecosystem fragmentation: a review. Conserv Biol. 1991; 5(1): 18–32.

[4] Smith JNM, Hellmann JJ. Population persistence in fragmented landscapes. Trends Ecol Evol. 2002; 17(9): 397–9.

[5] Fahrig L. Effects of habitat fragmentation on biodiversity. Annu Rev Ecol Syst. 2003; 34: 487–515.

[6] Peres CA. Synergistic effects of subsistence hunting and habitat fragmentation on Amazonian forest vertebrates. Conserv Biol. 2001; 15(6): 1490–505.

[7] Tabarelli M, Cardoso da Silva JM, Gascon C. Forest fragmentation, synergisms and the impoverishment of neotropical forests. Biodivers Conserv. 2004; 13(7): 1419–25.

[8] Treves A, Karanth KU. Human-carnivore conflict and perspectives on carnivore management worldwide. Conserv Biol. 2003; 17(6): 1491–9.

[9] Cardillo M, Purvis A, Sechrest W, Gittleman JL, Bielby J, Mace GM. Human population density and extinction risk in the world’s carnivores. PLoS Biol. 2004; 2(7): 909–14. 10.1371/journal.pbio.0020197 PMC449851 15252445

[10] Young JK, Olson KA, Reading RP, Amgalanbaatar S, Berger J. Is wildlife going to the dogs? Impacts of feral and free-roaming dogs on wildlife populations. Bioscience. 2011; 61(2): 125–32.

[11] Gompper ME. The dog-human-wildlife interface: assessing the scope of the problem In: Gompper ME, editor. Free-Ranging Dogs and Wildlife Conservation. Oxford: Oxford University Press; 2014 pp. 9–54.

[12] Vanak AT, Gompper ME. Dogs *Canis familiaris* as carnivores: their role and function in intraguild competition. Mammal Rev. 2009; 39(4): 265–83.

[13] Hughes J, Macdonald DW. A review of the interactions between free-roaming domestic dogs and wildlife. Biol Conserv. 2013; 157: 341–51.

[14] Craft ME, Vial F, Miguel E, Cleaveland S, Ferdinands A, Packer C. Interactions between domestic and wild carnivores around the greater Serengeti ecosystem. Anim Conserv. 2017; 20: 193–204.

[15] Vanak AT, Dickman CR, Silva-Rodríguez EA, Butler JRA, Ritchie EG. Top-dogs and under-dogs: competition between dogs and sympatric carnivores In: Gompper ME, editor. Free-Ranging Dogs and Wildlife Conservation. Oxford: Oxford University Press; 2014 pp. 69–93.

[16] Zapata-Ríos G, Branch LC. Altered activity patterns and reduced abundance of native mammals in sites with feral dogs in the high Andes. Biol Conserv. 2016; 193: 9–16.

[17] Duellman WE. Distribution patterns of amphibians in South America In: Duellman WE, editor. Patterns of Distribution of Amphibians: A Global Perspective. Baltimore: John Hopkins University Press; 1999 pp. 255–328.

[18] Knapp S. Assessing patterns of plant endemism in Neotropical uplands. Bot Rev. 2002; 68(1): 22–37.

[19] Young KR. Wildlife conservation in the cultural landscapes of the central Andes. Landsc Urban Plann. 1997; 38(3–4): 137–47.

[20] Hofstede R, Coppus R, Mena Vásconez P, Segarra P, Wolf J, Sevink J. El estado de conservación de los páramos de pajonal en el Ecuador. Ecotropicos. 2002; 15(1): 3–18.

[21] Sarmiento FO. Anthropogenic change in the landscapes of highland Ecuador. Geogr Rev. 2002; 92(2): 213–34.

[22] Goldstein I, Paisley S, Wallace R, Jorgenson JP, Cuesta F, Castellanos A. Andean bear-livestock conflicts: a review. Ursus. 2006; 17(1): 8–15.

[23] Parsons AW, Bland C, Forrester T, Baker-Whatton MC, Schuttler SG, McShea WJ, Costello R, Kays R. The ecological impact of humans and dogs on wildlife in protected areas in eastern North America. Biol Conserv. 2016; 203: 75–88.

[24] Myers N, Mittermeier RA, Mittermeier CG, da Fonseca GAB, Kent J. Biodiversity hotspots for conservation priorities. Nature. 2000; 403(333): 853–8. 10706275 10.1038/35002501

[25] Tirira DG. Mamíferos del Ecuador: Guía de Campo. Quito: Editorial Murciélago Blanco; 2007.

[26] Tirira DG, editor. Libro Rojo de los Mamíferos del Ecuador. Quito: Fundación Mamíferos y Conservación, Pontificia Universidad Católica del Ecuador, Ministerio del Ambiente; 2011.

[27] Ceballos G, Ehrlich PR. Mammal population losses and the extinction crisis. Science. 2002; 296 (5569): 904–7. doi: 10.1126/science.1069349 11988573 10.1126/science.1069349

[28] With KA, Crist TO. Critical thresholds in species' responses to landscape structure. Ecology. 1995; 76(8): 2446–59.

[29] Parsons JJ. The northern Andean environment. Mt Res Dev. 1982; 2(3): 253–64.

[30] Hess CG. "Moving up-moving down": agro-pastoral land-use patterns in the Ecuadorian Paramos. Mt Res Dev. 1990; 10(4): 333–42.

[31] Winckell A, Zebrowski C, Sourdat M. Las Regiones y Paisajes del Ecuador. Quito: Instituto Geográfico Militar; 1997.

[32] Baquero F, Sierra R, Ordóñez L, Tipán M, Espinosa L, Rivera MB, et al La Vegetación de los Andes del Ecuador. Quito: EcoCiencia, CESLA, Corporación EcoPar, Ministerio de Agricultura, Jatun Sacha, Instituto Geográfico Militar; 2004.

[33] Sarmiento FO. Breaking mountain paradigms: ecological effects on human impacts in man-aged Tropandean Landscapes. Ambio. 2000; 29(7): 423–31.

[34] Novaro AJ. *Pseudalopex culpaeus* (Molina, 1782). Mamm. Species. 1997; 558: 1–8.

[35] Sheffield SR, Thomas HW. *Mustela frenata* Lichtenstein, 1831. Mamm. Species. 1997; 570: 1–9.

[36] Peralvo MF, Cuesta F, van Manen FT. Delineating priority habitat areas for the conservation of Andean bears in northern Ecuador. Ursus. 2005; 16(2): 222–33.

[37] Tirira DG, González-Maya JF. Current state of knowledge of the least-known carnivore in South America: Colombian Weasel *Mustela felipei* in Colombia and Ecuador. Small Carniv Conserv. 2009; 41: 46–50.

[38] International Union for the Conservation of Nature (IUCN). The IUCN Red List of Threatened Species, Version 2013 2 IUCN Global Species Programme 2013 Available: http://www.iucnredlist.org/.

[39] Silva Rodríguez EA, Ortega-Solís GR, Jiménez JE. Conservation and ecological implications of the use of space by chilla foxes and free-ranging dogs in a human-dominated landscape in southern Chile. Austral Ecol. 2010; 35: 765–77.

[40] Smith DA. The spatial patterns of indigenous wildlife use in western Panama: Implications for conservation management. Biol Conserv. 2008; 141: 925–37.

[41] Zapata Ríos G, Urgilés C, Suárez E. Mammal hunting by the Shuar of the Ecuadorian Amazon: is it sustainable? Oryx. 2009; 43(3): 375–85.

[42] McGarigal K, Cushman SA, Neel MC, Ene E. 2002 FRAGSTATS: Spatial Pattern Analysis Program for Categorical Maps. The University of Massachusetts 2002. Available: http://www.umass.edu/research/fragstats.html/.

[43] Instituto Geográfico Militar (IGM). 2011 Cartografía Base del Ecuador a Escala 1:50.000. IGM 2011. Available: http://www.geoportaligm.gob.ec/.

[44] Silva-Rodríguez EA, Sieving KE. Domestic dogs shape the landscape-scale distribution of a threatened forest ungulate. Biol Conserv. 2012; 150:103–10.

[45] MacKenzie DI, Nichols JD, Lachman GB, Droege S, Royle JA, Langtimm CA. Estimating site occupancy rates when detection probabilities are less than one. Ecology. 2002; 83(8): 2248–55.

[46] MacKenzie DI. Modeling the probability of resource use: the effect of, and dealing with, detecting a species imperfectly. J Wildl Manag. 2006; 70(2): 367–74.

[47] Schuette P, Wagner AP, Wagner ME, Creel S. Occupancy patterns and niche partitioning within a diverse carnivore community exposed to anthropogenic pressures. Biol Conserv. 2013; 158: 301–12.

[48] Lucherini M, Reppucci JI, Walker RS, Villalba ML, Wurstten A, Gallardo G, et al Activity pattern segregation of carnivores in the high Andes. J Mammal. 2009; 90(6): 1404–9.

[49] Cozzi G, Broekhuis F, McNutt JW, Turnbull LA, Macdonald DW, Schmid B. Fear of the dark or dinner by moonlight? reduced temporal partitioning among Africa's large carnivores. Ecology. 2012; 93(12): 2590–9. 23431590 10.1890/12-0017.1

[50] Barea-Azcón JM, Virgós E, Ballesteros-Duperón E, Moleón M, Chirosa M. Surveying carnivores at large spatial scales: a comparison of four broad-applied methods. Biodivers Conserv. 2007; 16: 1213–30.

[51] Gerber BD, Karpanty SM, Kelly MJ. Evaluating the potential biases in carnivore capture–recapture studies associated with the use of lure and varying density estimation techniques using photographic-sampling data of the Malagasy civet. Popul Ecol. 2012; 54: 43–54.

[52] Burnham KP, Anderson DR. Model Selection and Multimodel Inference: A Practical Information-Theoretic Approach. New York: Springer; 2002.

[53] MacKenzie DI, Bailey LL. Assessing the fit of site-occupancy models. J Agric Biol Environ Stat. 2004; 9(3): 300–18.

[54] Hines JE. PRESENCE: Software to Estimate Patch Occupancy and Related Parameters. Patuxent Wildlife Research Center 2006 Available: http://www.mbr-pwrc.gov/software/presence.html/.

[55] Bailey LJ, Reid JA, Forsman ED, Nichols JD. Modeling co-occurrence of northern spotted and barred owls: accounting for detection probability differences. Biol Conserv. 2009; 142: 2983–9.

[56] MacKenzie DI, Bailey LL, Nichols JD. Investigating species co-occurrence patterns when species are detected imperfectly. J Anim Ecol. 2004; 73: 546–55.

[57] Lichstein JW, Simons TR, Shriner SA, Franzreb KE. Spatial autocorrelation and autoregressive models in ecology. Ecol Monogr. 2002; 72(3): 445–63.

[58] Legendre P, Legendre L. Numerical Ecology. Amsterdam: Elsevier; 1998.

[59] Wong DWS, Lee J. Statistical Analysis of Geographic Information with ArcView GIS and ArcGIS. Hoboken: John Wiley & Sons; 2005.

[60] R Core Team. R: A Language and Environment for Statistical Computing R Foundation for Statistical Computing 2013 Available: http://www.R-project.org/.

[61] Stasinopoulos DM, Rigby RA. Detecting break points in generalized linear models. Comput Stat Data Anal. 1992; 13(4): 461–71.

[62] Manor R, Saltz D. The impact of free-roaming dogs on gazelle kid/female ratio in a fragmented area. Biol Conserv. 2004; 119: 231–6.

[63] Vanak AT, Gompper ME. Interference competition at the landscape level: the effect of free-ranging dogs on a native mesocarnivore. J Appl Ecol. 2010; 47(6): 1225–32.

[64] Broquet T, Johnson A, Petit E, Thompson I, Burel F, Fryxell JM. Dispersal and genetic structure in the American marten, *Martes americana*. Mol Ecol. 2006; 15: 1689–97. doi: 10.1111/j.1365-294X.2006.02878.x 16629821 10.1111/j.1365-294X.2006.02878.x

[65] Ausband D, Moehrenschlager A. Long-range juvenile dispersal and its implication for conservation of reintroduced swift fox *Vulpes velox* populations in the USA and Canada. Oryx. 2009; 43(1): 73–7.

[66] Elbroch M, Wittmer HU, Saucedo C, Corti P. Long-distance dispersal of a male puma (*Puma concolor puma*) in Patagonia. Rev Chil Hist Nat. 2009; 82: 459–61.

[67] Cuesta F, Peralvo MF, van Manen FT. Andean bear habitat use in the Oyacachi river basin, Ecuador. Ursus. 2003; 14(2): 198–209.

[68] Ríos-Uzeda B, Gómez H, Wallace RB. Habitat preferences of the Andean bear (*Tremarctos ornatus*) in the Bolivian Andes. J Zool. 2006; 268: 271–8.

[69] Guénette JS, Villard MA. Thresholds in forest bird response to habitat alteration as quantitative targets for conservation. Conserv Biol. 2005; 19(4): 1168–80.

[70] Lindenmayer DB, Luck GW. Synthesis: thresholds in conservation and management. Biol Conserv. 2005; 124: 351–4.

[71] Andersen T, Carstensen J, Hernández-García E, Duarte CM. Ecological thresholds and regime shifts: approaches to identification. Trends Ecol Evol. 2009; 24(1): 49–57. doi: 10.1016/j.tree.2008.07.014 18952317 10.1016/j.tree.2008.07.014

[72] Radford JQ, Bennett AF. Thresholds in landscape parameters: occurrence of the white-browed treecreeper *Climacteris affinis* in Victoria, Australia. Biol Conserv. 2004; 117: 375–91.

[73] Betts MG, Forbes GJ, Diamond AW. Thresholds in songbird occurrence in relation to landscape structure. Conserv Biol. 2007; 21(4): 1046–58. doi: 10.1111/j.1523-1739.2007.00723.x 17650254 10.1111/j.1523-1739.2007.00723.x

[74] Lindenmayer DB, Fischer J, Cunningham RB. Native vegetation cover thresholds associated with species responses. Biol Conserv. 2005; 124: 311–6.

[75] Zuckerberg B, Porter WF. Thresholds in the long-term responses of breeding birds to forest cover and fragmentation. Biol Conserv. 2010; 143: 952–62.

[76] Swihart RK, Atwood TC, Goheen JR, Scheiman DM, Munroe KE, Gehring TM. Patch occupancy of North American mammals: is patchiness in the eye of the beholder? J Biogeogr. 2003; 30: 1259–79.

[77] Muntifering JR, Dickman AJ, Perlow LM, Hruska T, Ryan PG, Marker LL, et al Managing the matrix for large carnivores: a novel approach and perspective from cheetah (*Acinonyx jubatus*) habitat suitability modelling. Anim Conserv. 2006; 9: 103–12.

[78] Gehring TM, Swihart RK. Home range and movements of long-tailed weasels in a landscape fragmented by agriculture. J Mammal. 2004; 85(1): 79–86.

[79] Erlinge S, Sandell M, Brinck C. Scent-marking and its territorial significance in stoats, *Mustela erminea*. Anim Behav. 1982; 30(3): 811–8.

[80] Hutchings MR, White PCL. Mustelid scent-marking in managed ecosystems: implications for population management. Mammal Rev. 2000; 30(3–4): 157–69.

[81] Izor RJ, de la Torre L. A new species of weasel (*Mustela*) from the highlands of Colombia, with comments on the evolution and distribution of South American weasels. J Mammal. 1978; 59(1): 92–102.

[82] Villalba ML, Bernal N, Nowell K, Macdonald DW. Distribution of two Andean small cats (*Leopardus jacobita* and *Leopardus colocolo*) in Bolivia and the potential impacts of traditional beliefs on their conservation. Endanger Species Res. 2012; 16: 85–94.

[83] Sánchez F, Sánchez-Palomino P, Cadena A. Species richness and indices of abundance of medium-sized mammals in Andean forest and reforestations with Andean alder: a preliminary analysis. Caldasia. 2008; 30(1): 197–208.

[84] Balaguera-Reina SA, Cepeda A, Zárrate-Charry D, González-Maya JF. The state of knowledge of western mountain coati *Nasuella olivacea* in Colombia, and extent of occurrence in the Northern Andes. Small Carniv Conserv. 2009; 41: 35–40.

[85] Silva-Rodríguez EA, Sieving KE. Influence of care of domestic carnivores on their predation on vertebrates. Conserv Biol. 2011;25(4):808–15. doi: 10.1111/j.1523-1739.2011.01690.x 21658128 10.1111/j.1523-1739.2011.01690.x

[86] Silva-Rodríguez EA, Verdugo C, Aleuy OA, Sanderson JG, Ortega Solís GR, Osorio Zúñiga F, et al Evaluating mortality sources for the Vulnerable pudu *Pudu puda* in Chile: implications for the conservation of a threatened deer. Oryx. 2009; 44(1): 97–103.

[87] Silva Rodríguez EA, Ortega-Solís GR, Jiménez JE. Conservation and ecological implications of the use of space by chilla foxes and free-ranging dogs in a human-dominated landscape in southern Chile. Austral Ecol. 2010; 35: 765–77.

[88] Torres PC, Prado PI. Domestic dogs in a fragmented landscape in the Brazilian Atlantic Forest: abundance, habitat use and caring by owners. Braz J Biol. 2010; 70(4): 987–94. 21180903 10.1590/s1519-69842010000500010

[89] Instituto Ecuatoriano de Estadísticas y Censos (INEC). El Ecuador en Cifras: Población y Demografía. INEC 2014 Available: http://www.ecuadorencifras.gob.ec/.

[90] Josse C, Cuesta F, Navarro G, Barrena V, Cabrera E, Chacón-Moreno E, et al Ecosistemas de los Andes del Norte y Centro: Bolivia, Colombia, Ecuador, Perú y Venezuela Lim: Secretaría General de la Comunidad Andina, ECOBONA, CONDESAN, Programa BioAndes, EcoCiencia, NatureServe; 2009.

